# Therapeutic Applications of Stem Cell-Derived Exosomes

**DOI:** 10.3390/ijms25063562

**Published:** 2024-03-21

**Authors:** Omar Abdulhakeem Ahmed Yusuf Abdulmalek, Khaled Hameed Husain, Haya Khaled Ali Abdulla AlKhalifa, Mariam Masood Abdulkarim Bahrooz Alturani, Alexandra E. Butler, Abu Saleh Md Moin

**Affiliations:** 1 School of Medicine, Royal College of Surgeons in Ireland-Bahrain, Busaiteen 15503, Adliya, Bahrain; 18219136@rcsi.com (O.A.A.Y.A.); 20200259@rcsi.com (K.H.H.); 20200304@rcsi.com (H.K.A.A.A.); 20200908@rcsi.com (M.M.A.B.A.); 2 Research Department, Royal College of Surgeons in Ireland-Bahrain, Busaiteen 15503, Adliya, Bahrain; amoin@rcsi.com

**Keywords:** stem cells, exosomes, microRNAs, diabetic microvascular complications, precision medicine

## Abstract

Exosomes are extracellular vesicles of endosomal origin, ranging from 30 to 150 nm in diameter, that mediate intercellular transfer of various biomolecules, such as proteins, lipids, nucleic acids, and metabolites. They modulate the functions of recipient cells and participate in diverse physiological and pathological processes, such as immune responses, cell–cell communication, carcinogenesis, and viral infection. Stem cells (SCs) are pluripotent or multipotent cells that can differentiate into various cell types. SCs can also secrete exosomes, which exhibit remarkable therapeutic potential for various diseases, especially in the field of regenerative medicine. For example, exosomes derived from mesenchymal stem cells (MSCs) contain proteins, lipids, and miRNAs that can ameliorate endocrine disorders, such as diabetes and cancer. Exosomes from SCs (sc-exos) may offer similar advantages as SCs, but with reduced risks and challenges. Sc-exos have lower tumorigenicity, immunogenicity, and infectivity. They can also deliver drugs more efficiently and penetrate deeper into tissues. In this review, we provide an overview of the recent advances in sc-exos and their therapeutic applications in various diseases, such as diabetes and cancer. We also elucidate how the biological effects of sc-exos depend on their molecular composition. We also address the current challenges and future directions of using sc-exos.

## 1. Introduction

Stem cells (SCs) are unspecialized cells that can differentiate into various cell types and have a capacity for self-renewal [1]. SCs can be sourced from both adult cells and embryos, and their therapeutic applications continue to grow in the field of regenerative medicine. The secretory ability of SCs is of increasing importance, as their ability to release various soluble factors and exosomes is now thought to play a more critical role in tissue regeneration [2]. Exosomes are extracellular vesicles (EVs) that are secreted by a majority of cell types and are found dispersed in various body fluids, such as the blood, breast milk, and cerebrospinal fluid (CSF) [3]. EVs are cell-secreted structures that are enclosed by a lipid bilayer membrane, vary in size, cargo, and protein markers, and are crucial for cell-to-cell communication. Initially, exosomes were thought to be of minimal use and functioned as “rubbish bins’’ to dispose of cellular waste products, but recent research has revealed the various functions they are capable of, including drug-carrying abilities and altering pathological processes, particularly in the mechanistic aspects of cancer (such as metastasis and drug resistance) [4,5].

Exosomes can be derived from various types of SCs; however, a widely researched source is mesenchymal SCs (MSCs). MSCs are a class of multipotent SCs with various biological and functional properties. MSCs are capable of multidirectional differentiation and self-renewal and are derived from various tissues, including the bone marrow, umbilical cord, adipose tissue, and muscle [6]. The applications of MSCs continue to grow in the field of regenerative medicine, where much of the literature has focused on their use in neuropathologies, ischemic heart disease, diabetes mellitus (DM), and orthopedic diseases [7]. Another type of stem cell is human embryonic stem cells (hESCs), which possess pluripotent differentiating abilities and have comparable characteristics to that of MSCs [8]. These cells can be sourced from the inner cell mass of a blastocyst and, given their altered proteome compared to MSCs; the resultant exosomes are likely to have differing proliferation and self-renewal capabilities due to their altered cargo [9]. Similar to hESCs, induced pluripotent stem cells (iPSCs) are another class of SCs that have been reprogrammed from a differentiated cell form to an embryonic stem cell-like state and possess the ability to secrete exosomes, and the resultant bioactive material has proven useful in tissue repair due to a variety of pathologies [10].

Stem cell-derived exosomes (sc-exos) are now thought to be largely responsible for the paracrine signaling processes that take place in wound healing and tissue repair [11,12]. Moreover, sc-exos contain numerous cytokines: transforming growth factor beta (TGF-ß), vascular endothelial growth factor (VEGF), interleukin (IL)-6, IL-10, various chemokines, such as chemokine (C-C motif) ligand 7 (CCL7) and chemokine (C-X-C motif) ligand 2 (CXCL2), and many more, which are involved in regulating signaling responses important for immunomodulation, cell proliferation, and growth [13,14]. Sc-exos also contain bioactive molecules that are internalized by cells, such as fibroblasts, endothelial cells, and keratin-forming cells [15]. These bioactive molecules include various proteins, lipids, microRNAs (miRNAs), and non-coding RNAs (ncRNAs), which are fundamentally involved in tissue repair and regeneration [16]. The internalization of these biomolecules into the aforementioned recipient cells is facilitated by the many membrane-bound molecules found on the lipid bilayers of these exosomes, making this an essential property of these vesicles [17]. The benefits of sc-exos in regenerative medicine are similar to that of SCs; however, many of the risks are mitigated. Because this therapy is cell-free, the cancer risk is significantly lowered since sc-exos cannot replicate. Additionally, exosomes have a diminished immunogenic response, and, given their smaller size and simplicity compared to cells, they are simpler to isolate and preserve [18].

Sc-exos clearly hold great potential in the field of regenerative medicine and may, in the future, serve as a substitute for SCs, given that many of the associated risks are diminished. Therefore, this review summarizes and analyzes the available literature directed towards current knowledge and future applications of sc-exos in treating a variety of pathologies, as well as giving consideration to future challenges that may arise.

## 2. Biogenesis, Composition, and Signaling Mechanisms of Exosomes

### 2.1. Biogenesis of Exosomes

#### 2.1.1. Early Sorting Endosomes

**Figure 1:**
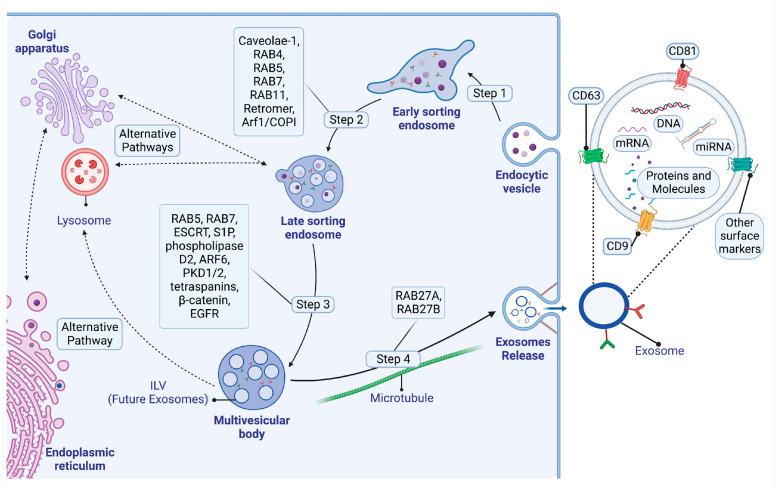
Biogenesis of exosomes. The chart depicts the transformation of endosomes into early-sorting endosomes (ESEs) (Step 1), their progression to late-sorting endosomes (LSEs) (Step 2), and the subsequent journey through the Endosomal Sorting Complex Required for Transport (ESCRT) and ESCRT-independent pathways to form multivesicular bodies (MVBs) (Step 3). Subsequently, with the assistance of Ras-related protein Rab-27A (RAB27A) and RAB27B, they are transported along microtubules for exocytosis (Step 4). These exosomes typically comprise Cluster of Differentiation (CD)63, CD81, CD9, DNA fragments, messenger RNA (mRNA), microRNA (miRNA), proteins, molecules and other components.

Exosome biogenesis starts within the endosomal system, specifically with early sorting endosomes (ESEs), which serve as the primary sites for organizing membrane-related cargo and facilitating solute transfer, as visually represented in Figure 1. Diverse receptors, encompassing nutrient transporters (such as various members of the glucose transporter family of receptors), lipoprotein receptors, and amino acid transporters, alongside ion channels and cell signaling receptors, can be present within an ESE. ESEs originate from the fusion of primary endocytic vesicles, essentially establishing themselves as fundamental endocytic vesicles [19].

Two primary mechanisms are involved in the genesis of endocytic vesicles: clathrin-mediated endocytosis (CME) and clathrin-independent endocytosis (CIE). ADP-ribosylation factor 6 (ARF6) and clathrin-independent carriers (CLIC)/glycosylphosphatidylinositol-enriched endocytic compartment (GEEC)-dependent pathways play integral roles in the CIE pathway. These pathways ultimately converge to facilitate the engulfment of molecules through phagocytosis [20]. Additionally, the CME pathway entails a series of sequential steps, encompassing nucleation, cargo selection, assembly of clathrin coats, vesicle scission, and uncoating [21].

ESEs exhibit two distinct structural forms, those that contain a tubular structure and those that contain a vacuole structure, and each is characterized by unique physiological functions and composition. Within the tubular structure, key molecules such as caveolae-1, GTPases (Ras-related protein 4 (Rab4), Rab5, Rab7, Rab11) [22,23], retromer and ADP-ribosylation factor 1 (ARF1)/coat protein complex I are present. Interestingly, retromer and caveolae-1 play crucial roles in sorting cargo destined for various targets [24]. These molecules facilitate the organization of cargos, including proteins, dissociated ligands, and solutes acquired from bodily fluids within the tubular vesicles [20].

#### 2.1.2. Late-Sorting Endosomes

The vacuole structure goes on to form the late-sorting endosome (LSE), as illustrated in Figure 1, though the underlying mechanism is yet to be fully understood. Research highlights that, prior to LSE development, Rab5 facilitates the recruitment of Rab7, therefore forming a transient heterozygous endosome. Subsequently, Rab5 is converted into its guanosine diphosphate (GDP) form and detaches with its effector. Meanwhile, a vacuole structure emerges, solely featuring Rab7, which continuously recruits cargo. Simultaneously, its membrane surface begins an inward budding process, thereby leading to the formation of small intraluminal vesicles (ILVs) [20,23,25].

#### 2.1.3. The Endosomal Sorting Complex Required for Transport (ESCRT) Pathway

Exosomes originate from late endosomes, leading to the creation of ILVs within large multivesicular bodies (MVBs) [26], as visually represented in Figure 1. During ILV formation, specific proteins are integrated into the budding membrane, while cytosolic components are encapsulated within ILVs. Subsequently, ILVs are released into the extracellular space as “exosomes” following fusion with the plasma membrane [27,28].

The formation of ILVs within late endosomes is dependent on the presence of the late endosomal sorting complex required for transport (ESCRT). Comprising four distinct complexes (ESCRT-0 to ESCRT-III), and the ESCRT system collaborates to facilitate essential processes such as vesicle budding, protein cargo sorting, and MVB formation. The initiation of the ESCRT involves the selective recognition and sequestration of ubiquitinated proteins to specific regions of the endosomal membrane through the ubiquitin-binding subunits of ESCRT-0 [29,30].

Following interaction with ESCRT-I and II complexes, the combined complex associates with ESCRT-III, a protein complex pivotal for facilitating the budding processes. Subsequently, the ESCRT-III complex facilitates the cleavage of buds, resulting in the generation of ILVs within MVBs. Ultimately, the ESCRT-III complex dissociates from the MVB membrane, driven by the sorting protein Vps4, which supplies the necessary energy for separation [29].

Although the role of the ESCRT in exosome release remains a topic of ongoing debate, extensive research has highlighted the presence of diverse ESCRT components and ubiquitinated proteins within exosomes obtained from various cell types. The exosomal protein Alix, known for its association with ESCRT proteins, actively participates in processes such as endosomal membrane budding, abscission, and cargo selection within exosomes through its interaction with syndecan. These significant findings emphasize the crucial involvement of the ESCRT in exosome biogenesis, highlighting its functional role in this cellular process [31].

#### 2.1.4. The ESCRT-Independent Pathway

Evolving research has revealed that MVB and intralumenal vesicle (ILV) formation are not solely dependent on the ESCRT complex. For instance, it is widely recognized that lipids play a crucial role in shaping and structuring cell membranes. Stuffers et al. have reported the connection between the release of exosomes originating from mouse oligodendrocytes and neutral sphingomyelinase, an enzyme responsible for generating ceramide [32]. The study highlighted that introducing synthetic ceramide can facilitate MVB budding by metabolism into sphingosine 1 phosphate (S1P), which subsequently binds to the S1P receptor on MVBs, stimulating ILV production [33]. Furthermore, an interesting discovery was made regarding phospholipase D2, which was identified as the effector protein associated with the small GTPase ADP-ribosylation factor 6 (ARF6). ARF6 plays a pivotal role in the budding process of MVBs and the consequent generation of exosomes [34]. Moreover, Maxxeo et al. have reported the role of diacylglycerol kinase α in enhancing the maturation and subsequent release of MVBs, achieved through the regulation of polycystin (PKD)-1/2 activation [35].

Tetraspanins also participate in the ESCRT-independent process of generating exosomes. These proteins are recognized for their role in structuring microdomains within the cellular membrane. This organization occurs as tetraspanins cluster together and engage with proteins situated both in the membrane and in the internal signaling network of the cell. These specialized zones, known as tetraspanin-enriched microdomains, serve as effective platforms for conveying cargo [36]. Notably, the collaboration between tetraspanins CD9 and CD82 and e-cadherin leads to the enhancement of β-catenin exosomal secretion [37]. An additional ESCRT-independent pathway works through the epidermal growth factor receptor (EGFR). A recent study highlighted that EGFR was present in exosomes derived from the serum of cancer patients, indicating that the ESCRT complex might not exclusively oversee the generation of exosomes. Additionally, the study revealed that EGFR-lacking ubiquitin molecules could be internalized by cells during periods of serum deprivation. Remarkably, these EGFR molecules were observed to colocalize with CD63-positive MVBs formed under the influence of Rab31 [38].

### 2.2. The Structure and Composition of Exosomes

Exosomes possess a remarkable degree of complexity and versatility, containing a diverse array of components that encompass 194 lipids, 4400 proteins, 764 miRNAs, and 1639 messenger RNAs (mRNA) [39,40]. Notably, tetraspanins such as CD9, CD63, CD81, and CD82 are among the prominent proteins abundantly present in exosomes, playing significant roles in processes including cell penetration, invasion, and fusion. Additionally, heat shock proteins (HSP70 and HSP90) found within exosomes contribute to stress responses, including antigen binding and presentation. Furthermore, specific proteins such as heat shock protein 70 (HSP70, CD81, tumor susceptibility gene 101, and CD63, known for their abundance in MVBs, are commonly utilized as markers to identify and characterize exosomes [41,42].

Exosomal components do not remain constant but, rather, are continuously changing depending upon the condition of the cell [43]. For instance, a particular microRNA, microRNA (miR)-1246, was markedly increased in the exosomes of various cancer types, including melanoma, glioma, colorectal, breast, renal, oral, laryngeal, pancreatic, and ovarian cancers. Another study highlighted the efficiency of miR-1246 in diagnosing colorectal cancer (CRC), demonstrating 100% sensitivity and 80% specificity [44]. Notably, miR-1246 outperforms traditional CRC tests such as carcinoembryonic antigen (CEA) and cancer antigen 19-9 (CA19-9), both of which exhibit considerably lower sensitivity and specificity [45]. Studies have identified specific proteins encapsulated within exosomes associated with breast cancer diagnosis and prognosis. A meta-analysis reported significantly higher levels of cargo molecules human epidermal growth factor receptor 2 (HER2), CD47, Del-1, miR-1246, and miR-21) in small EVs (sEVs) from breast cancer patients compared to healthy controls and those with benign breast tumors [46]. Certain proteins, such as HER2, kinase insert domain receptor (KDR), CD49d, C-X-C motif chemokine receptor 4 (CXCR4), and CD44, were found to be associated with tumor recurrence or distant organ metastasis [47]. Some exosomal components can be glycosylated, depending on the cell of origin. As the glycosylation patterns of exosomes reflect those of their cells of origin, this leads to marked differences between exosomes derived from tumor versus non-tumor cells [48]. Exploiting these differences, exosomal glycosylation patterns can be utilized as highly specific and readily detectable biomarkers for diseases such as cancer [49].

In the context of lung cancer, a study focused on non-small-cell lung cancer (NSCLC) demonstrated that NSCLC-derived sEVs (NSCLC-sEVs) abundantly express specific N-glycan structures, such as core fucosylated, biantennary, and triantennary N-glycans. Additionally, the study found that NSCLC-sEVs express the integrin α6β4 and that the N-glycans on the integrin α6 subunit exhibit NSCLC-specific characteristics [50]. By contrast, the N-glycan profiles of small-cell lung cancer (SCLC)-derived sEVs showed brain-related characteristics, which can be attributed to the neuroendocrine properties of SCLC cells. This difference in glycosylation patterns is indicative of the heterogeneity in the properties of the cells of origin and highlights the potential of protein N-glycosylation in sEVs as a means to identify the originating lung cancer types [50]. By targeting the protein N-glycosylation of cancer sEVs, it may be possible to detect and distinguish specific cancer types and their origins, leading to the development of personalized therapeutic strategies.

Overall, exosomal glycosylation patterns serve as potential diagnostic biomarkers for certain diseases. The analysis of exosomal glycosylation offers valuable insights into the presence of diseases in different organ systems. These findings highlight the potential of exosomal glycosylation as a non-invasive diagnostic approach with broad clinical applications [50].

### 2.3. Cellular Uptake and Signaling of Exosomes: Mechanisms and Implications

#### 2.3.1. Uptake and Internalization of Exosomes by Cells

The uptake of exosomes is a rapid process and is affected by temperature, with low temperature decreasing uptake. Moreover, internalization is accomplished through many common endocytic pathways encompassing macropinocytosis, phagocytosis, lipid raft or clathrin-mediated endocytosis (CME) [51].

#### 2.3.2. Clathrin-Mediated Endocytosis

CME is characterized by the assembly of transmembrane receptors and ligands. This mechanism involves the formation of clathrin-coated vesicles through the participation of a triskelion scaffold known as clathrin. Once internalized, the vesicles undergo uncoating and subsequently fuse with endosomes, facilitating their integration into cellular pathways [52]. The significance of CME in exosome uptake is evident across a range of cell types. Epithelial cells, hepatocytes, cardiomyocytes, macrophages, neural cells, as well as colon and ovarian tumor cells, all rely on factors essential for CME to facilitate exosome internalization [53,54,55,56]. This highlights the involvement of this pathway in physiological and pathological contexts. Moreover, cancer cells exhibiting overexpression of the transferrin receptor, a cargo molecule associated with CME, demonstrate enhanced uptake of exosomes [51], indicating that alterations in receptor expression levels may modulate the efficiency of exosome internalization in cancer cells.

#### 2.3.3. Lipid Raft-Mediated Endocytosis

Lipid raft-mediated membrane endocytosis is a crucial mechanism that plays a significant role in directing cargo towards the early endosome and influences exosome uptake [57]. Lipid rafts possess a specific architectural composition characterized by detergent-resistant membrane microdomains enriched in sphingolipids, cholesterol, and glycosylphosphatidylinositol (GPI)-anchored proteins [58].

Interestingly, it has been observed that the inhibition of complex lipid metabolism can impact exosome uptake through lipid rafts. For instance, the use of methyl-β-cyclodextrin has been shown to disrupt intracellular cholesterol transport, leading to a reduction in exosome uptake in breast cancer cells [59]. Furthermore, studies have demonstrated that pretreating exosome-producing cells with a sphingolipid inhibitor impaired their uptake by dendritic cells [60]. Additionally, pretreatment of tumor cells with flippin, a molecule known to bind cholesterol and form ultrastructural aggregates, also resulted in a diminished uptake of exosomes [61,62]. These findings highlight the interplay between lipid raft-mediated endocytosis and exosome uptake, which may help to guide potential therapeutic targets.

#### 2.3.4. Phagocytosis

Phagocytosis is widely recognized for its ability to engulf various types of particles, including bacteria. However, this cellular process is not limited to larger particles and can also involve the engulfment of smaller entities like exosomes [63]. Immune cells, particularly dendritic cells and macrophages, predominantly utilize phagocytosis to internalize exosomes [56,64].

The phagocytic process commences with the deformation of the cell membrane, which encircles the extracellular particles forming phagosomes [63]. Subsequently, the internalized cargo is directed towards lysosomes. Notably, the enzymes phosphatidylinositol-3-kinase (PI3K) and phospholipase C (PLC) play crucial roles in facilitating phagosome closure [56,64].

#### 2.3.5. Macropinocytosis

Macropinocytosis is an important cellular process that involves the utilization of actin-driven lamellipodia to initiate the inward invagination of the plasma membrane, leading to the formation of intracellular compartments called macropinosomes [65]. Recent studies have revealed that macropinocytosis is tightly regulated by various factors, including cholesterol-mediated recruitment of the Rac Family Small GTPase 1 (Rac1) GTPase, the function of Na^+^/H^+^ exchangers and, in certain cases, dynamin [66]. Interestingly, macropinocytosis also plays a significant role in the internalization of exosomes. Specifically, in HeLa cells [67], certain subsets of microglial cells [68] and to some extent in epithelial cells [69], the uptake of exosomes relies on macropinocytosis, which is influenced by the activity of Na^+^/H^+^ exchangers [68]. Furthermore, research has demonstrated that rat sarcoma virus (RAS)-mediated macropinocytosis facilitates the uptake of engineered exosomes designed to target oncogenic kirsten-RAS [70]. These findings contribute to our understanding of the diverse functions and regulatory mechanisms associated with macropinocytosis and its role in exosome uptake and internalization.

#### 2.3.6. Exosome-Mediated Intercellular Communication

Exosomes have recently gained recognition as a novel mechanism of intercellular communication, a discovery rooted in the observation that these vesicles, released by parent cells, can engage and influence target cell behavior. This interaction is primarily facilitated through the transmission of genetic material, which occurs via two main pathways: receptor–ligand interactions and direct internalization. In the case of receptor–ligand interactions, exosomes display surface molecules that can bind to specific receptors on target cells, initiating signaling cascades that modulate cellular functions. For instance, TGF-β-laden exosomes can activate the suppressor of mothers against decapentaplegic (SMAD) signaling pathway in recipient cells, influencing cell proliferation [71]. Alternatively, direct internalization allows the genetic content within exosomes, including functional proteins, RNAs, mRNA, miRNA, and transcription factors, to be transferred to the target cell’s cytoplasm. This can lead to the activation of signaling pathways such as the mitogen activated protein kinase (MAPK)/extracellular signal-regulated kinase (ERK) and PI3K/Protein kinase B (Akt) pathways, which play crucial roles in cell survival, proliferation, and differentiation [72,73]. Furthermore, the delivery of miRNAs by exosomes can modulate gene expression in recipient cells by affecting mRNA stability and translation, an example being the regulation of *PTEN* expression by exosomal miR-21, which activates the PI3K/Akt pathway [74]. The bioactive molecules present in exosomes thus impact target cells through various means, including the transfer of activated receptors and the induction of epigenetic changes, underlining the complexity and specificity of exosome-mediated communication [75].

## 3. Sources of Stem Cell-Derived Exosomes: Molecular Traits, Functionality, and Therapeutic Implications

The exploration of exosome biogenesis from stem cells has come to the forefront of scientific inquiry, revealing promising avenues for understanding cellular communication and potential therapeutic strategies. Stem cells, with their inherent ability to differentiate into various cell types, serve as a rich source of exosomes. This section explores processes of exosome production from stem cells, examining the molecular characteristics, synthesis regulation, and the multifaceted roles these vesicles play in physiological and pathological conditions. It further explores the field of exosome-based therapies, showcasing the therapeutic potential of exosomes derived from a diverse array of stem cells (Table 1).

**Figure 2:**
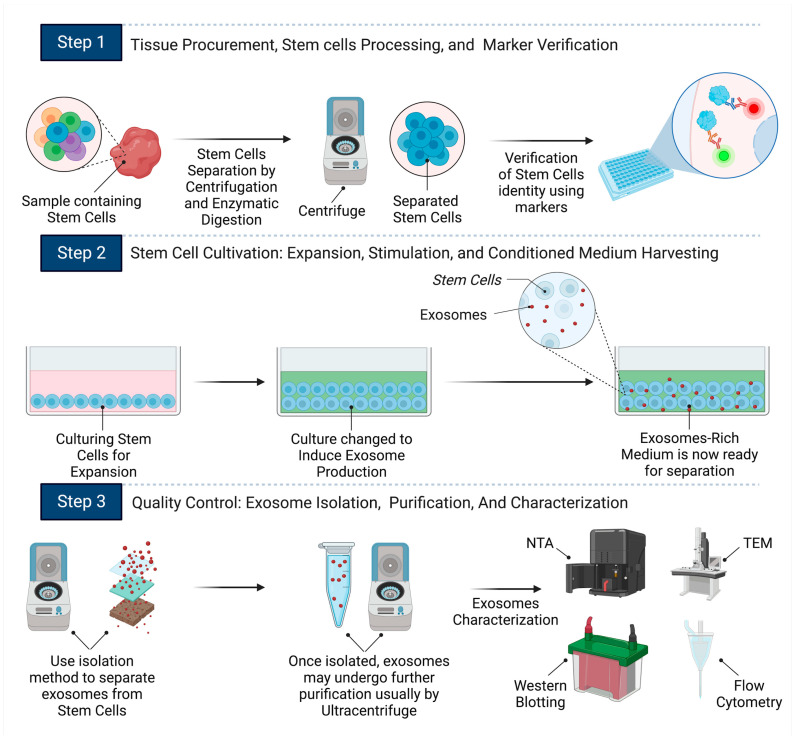
Comprehensive Flowchart of Exosome Production from stem cells. Beginning with tissue biopsy and proceeding through stem cell identification, cultivation, exosome induction, purification, characterization, and concluding with storage preparation for therapeutic or diagnostic applications. TEM: transmission electron microscopy; NTA: nanoparticle tracking analysis.

The flowchart in Figure 2 summarizes the journey of exosome production from stem cells, starting from tissue biopsy, through the stages of stem cell identification, cultivation and exosome induction, to the purification, characterization, and storage preparation of exosomes. Subsequent sections highlight the unique properties and therapeutic prospects of exosomes derived from these stem cell sources, paving the way for innovative treatment strategies across a range of medical conditions.

### 3.1. Exosomes from Adipose-Derived Stem Cells

Adipose-derived stem cells (ADSCs) are widely acknowledged for their easily accessible tissue sources and their diverse differentiation capabilities. They are multipotent stem cells that are able to transform into a variety of cell types, including adipocytes, osteoblasts, chondrocytes, and myocytes. As a result, exosomes from ADSCs are invaluable for tissue repair purposes, notably in bone fracture healing and limb ischemia [76,77].

To extract ADSCs, an adipose tissue sample is first acquired through liposuction. These cells are then separated from the adipose tissue using an enzymatic process with collagenase. After this digestion, the tissue is centrifuged, separating out the ADSCs. To confirm the identity of ADSCs, surface markers such as CD29, CD44, CD73, CD90, and CD105 are typically used as positive identifiers, while hematopoietic markers like CD31, CD34, and CD45 are used as negative identifiers. Once isolated, ADSCs are cultivated in specialized growth mediums and, over time, release exosomes into the medium. The exosome-rich medium can then be harvested and centrifuged to remove residual cells and debris. An ultracentrifugation process helps pellet the exosomes, which are resuspended and further refined using techniques like size-exclusion chromatography, ensuring a high-quality exosome yield [76,78,79], as illustrated in Figure 2.

Exosomes derived from ADSCs typically fall within a size range of 30–200 nm. They exhibit a distinctive cup-like shape when viewed under transmission electron microscopy (TEM). Research focusing on these exosomes in the context of lower limb ischemia identified the presence of exosomal markers CD9, CD63, and CD81, while markers GM130 and β-tubulin were found to be absent [78,79]. Interestingly, a recent study has highlighted the role of the microRNA miR-125b-5p, found in ADSC-exos, in repairing muscle tissue damaged by ischemia. MiR-125b-5p acts as a key regulator under ischemic conditions, primarily by targeting and modulating the activity of a specific molecule known as alkaline ceramidase 2 (ACER2). ACER2 has been linked to the increased production of ROS when overexpressed, as observed in models of diabetic hindlimb ischemia. Consequently, the modulation of ACER2 by miR-125b-5p suggests a potential therapeutic pathway for treating muscle injuries in ischemic, and especially diabetic, patients [76].

Furthermore, introducing miR-125b-5p into C2C12 cells (a myocyte variant) stimulates cell growth and promotes cell movement. This effect is particularly significant as it directly counters the harmful overexpression of ACER2, providing a foundation for miR-125b-5p’s potential as a treatment strategy for ischemic muscle injury in diabetic patients through targeting ACER2 [76].

Further, ADSC-exos promote the growth and movement of C2C12 cells and boost angiogenesis in human umbilical vein endothelial cells (HUVECs). Laboratory tests on living organisms have shown that ADSC-exos can safeguard ischemic skeletal muscle, expedite muscle injury healing, and foster vascular regeneration. The pivotal molecule in these processes appears to be miR-125b-5p [78]. Another investigative study exploring the influence of ADSC-exos on nonunion bone fracture healing in diabetic rats found that ADSC-exos notably advanced the bone healing process. This enhancement might be driven by activation of the Wnt3a/β-catenin signaling pathway, which promotes osteogenic differentiation in bone marrow-derived stem cells (BMSCs). However, the exact mechanism and the precise exosomal component underlying this has yet to be delineated [79].

### 3.2. Exosomes from Bone Marrow-Derived Mesenchymal Stem Cells

BMSCs share the property of multipotency with other MSCs, enabling their differentiation into diverse mesodermal cell lineages. Obtaining a bone marrow sample involves a painful and invasive aspiration [80], often requiring a considerable sample volume, typically ranging from 20 to 50 mL [81]. Recent advancements have substantially minimized this requirement to approximately 6 mL [82]. This advancement is attributable to an extraction method known as red blood cell (RBC) lysis, which increases efficiency by enabling the collection of the same, if not greater, amounts of MSCs with a substantially lower blood marrow volume and, moreover, the time to generate the therapeutic range of MSCs from the aspirate was the same across the high and low volume donors [82]. RBC lysis is a faster and more easily standardized technique which can isolate MSCs by using ammonium chloride to break down RBCs whilst not affecting the composition of stem cells [83]. From a patient standpoint, studies have shown that low-volume aspirates are associated with fewer complications, such as minimal hemoglobin loss [84]. Furthermore, this offers a more efficient methodology to generate more MSCs.

Once the bone marrow is aspirated, typically from the iliac crest or metaphysis of the distal femur and proximal tibia, the isolation of BMSCs involves enzymatic digestion followed by centrifugation to separate them from other cell types. Distinctive surface markers, specifically high CD73, CD90, and CD105 expressions and low CD34 and CD45 expressions, serve to confirm the identity of BMSCs. After culturing BMSCs, exosomes can be extracted for further analysis [85], as illustrated in Figure 2.

Isolated exosomes have been characterized using nanoparticle tracking analysis (NTA). These BMSC-derived exosomes (BMSC-exos) had a diameter of 30 to 100 nm, the average being 90 nm. TEM revealed their discoid morphology. The BMSC-derived exosomes were found to strongly express exosome markers (CD63, CD9, and TSG101), together with increased levels of miR-96 [86,87].

An in vivo rat study investigated the effects of BMSC-derived exosomes on doxorubicin-induced myocardial toxicity. Echocardiography demonstrated enhanced cardiac systolic and diastolic function and reduced cardiac injury in rats subjected to doxorubicin, owing to the administration of BMSC-exos via an injection into a tail vein. BMSC-exos effectively mitigated inflammatory responses brought about by doxorubicin-induced myocardial toxicity, as evidenced by decreased levels of proinflammatory cytokines (tumor necrosis factor alpha (TNF-α), interleukin 1 beta (IL-1β) and IL-6) and collagen fibers, both of which are typically linked to doxorubicin-induced toxicity [87].

This protective effect was attributed to the content of miR-96 within BMSC-exos. Overexpression of miR-96 correlated with improved cardiac function, reduced oxidative stress, inflammation, and myocardial fibrosis. Conversely, inhibiting miR-96 negated the protective effects of BMSC-exos. This study highlighted the Rac1/nuclear factor (NF)-κB signaling pathway as a crucial mechanism through which miR-96 exerts its protective effects. Doxorubicin-induced cardiotoxicity upregulated Rac1 and NF-κB signaling, both of which were subsequently downregulated following BMSC-exos treatment. These findings suggest that miR-96 directly inhibits Rac1. Furthermore, inhibition of Rac1 resulted in decreased levels of proteins associated with the NF-κB signaling pathway, a major regulator of inflammation that can promote apoptosis in doxorubicin-induced cardiotoxicity. Therefore, the study purported that miR-96 alleviates doxorubicin-induced myocardial toxicity by inhibiting Rac1 and by subsequently downregulating the downstream NF-κB signaling pathway [87].

A separate investigation found that miR-221, present in higher concentrations within exosomes derived from diabetic BMSCs, plays a pivotal role in the bone–fat imbalance observed in diabetes. By targeting and downregulating the *RUNX2* gene, a transcription factor essential for osteoblast differentiation and skeletal morphogenesis, miR-221 suppresses osteogenesis while promoting adipogenesis. This imbalance leads to reduced bone mass and increased marrow fat accumulation. Targeting miR-221 is, therefore, a promising therapeutic avenue to rectify the bone–fat imbalance associated with diabetes [86].

### 3.3. Exosomes from Hair Follicle-Derived Mesenchymal Stem Cells

Hair follicle-derived mesenchymal stem cells (HFMSCs) have much in common with other MSCs. They can transform into various mesodermal cell types, mainly adipocytes, osteocytes, and chondrocytes [88]. HFMSCs can also differentiate into myocytes [89] and red blood cells [90].

After their isolation, as detailed in [88], HFMSCs are recognized using specific surface markers including CD29, CD44, CD73, CD90, CD105, and CD166. HFMSCs do not usually express hematopoietic markers such as CD31, CD34, and CD45 or keratinocyte markers like cytokeratin 15 (CK15) [90]. From the subsequent culture of HFMSCs, exosomes can be collected for further analysis [91], as illustrated in Figure 2.

Characterization of these exosomes using NTA recorded a diameter between 100 nm and 400 nm. Cryo-electron microscopy showed them to be round in shape. Markers present in the HFMSC-derived exosomes (HFMSC-exos) included CD13, CD9, CD63, CD105, CD81, CD29, CD44, and CD49e; they also expressed stage-specific embryonic antigen (SSEA)-4, a protein notable for promoting stem cell characteristics [91].

Markers found on these EVs help to explain their functions. For example, markers like CD13 and CD105 are associated with stem-like and restorative qualities, while adhesion markers such as CD29, CD44, and CD49e help EVs to bind to sites of injury. Tissue factor suggests early involvement in wound recovery. While this study underscores the role of HFMSC-exos in wound care, it also found that these EVs could decrease oxidative stress in dermal fibroblasts and boost their metabolic activity, but only under specific cytotoxic hyperglycemic conditions [91].

Only limited research exists on HFMSC-exos, leaving a critical knowledge gap; therefore, more research is crucial in this area [91].

### 3.4. Exosomes from Induced Pluripotent Stem Cells

Induced pluripotent stem cells (iPSCs) are capable of differentiating into the three primary germ layers: endoderm, mesoderm, and ectoderm. The Nobel Prize laureate Dr. Shinya Yamanaka first described these cells in 2006 and sparked significant interest within the scientific community due to their greater versatility compared to MSCs, which differentiate solely into mesodermal tissues [92,93].

The creation of iPSCs begins with the incorporation of specific transcription factors (TFs), namely Octamer-binding transcription factor 4 (Oct4), SRY (sex determining region Y)-box 2 (Sox2), Krüppel-like factor 4 (Klf4), and the myelocytomatosis oncogene (c-Myc), into somatic cells, such as fibroblasts. Typically, viral vectors are utilized to facilitate the entry of these TFs into the cell’s nucleus. Within the nucleus, the TFs latch onto distinct DNA regions, thus activating pluripotency genes like *OCT4, SOX2, NANOG, KLF4*, and *c-MYC*. The activation of these genes is necessary for preserving the undifferentiated state and inherent qualities of stem cells. This initiation is pivotal in the metamorphosis of somatic cells to a pluripotent state. Upon reprogramming, these cells proliferate, forming colonies reminiscent of embryonic stem cells in both morphology and growth attributes [92,93].

Subsequently, iPSCs can undergo cultivation to stimulate cell growth and exosome synthesis. The confirmation of iPSCs is based upon the expressions of specific markers, such as Oct3/4A, Nanog, Sox2, reduced expression gene 1 (Rex1), and placental alkaline phosphatase; further, they show expressions of CD49e, CD29, CD105, and CD117, which are all markers related to adhesion and growth factor receptors. Exosomes can be isolated from the cell culture using ultracentrifugation [92,93].

The procured exosomes display a mean diameter of 143.5 nm, as determined by NTA, and possess a distinctive cup-shaped structure, evident on TEM. Exosomes positively express the exosome markers CD9 and Tumor Susceptibility Gene 101 (TSG101), along with the iPSC-specific marker SSEA-1. Intriguingly, these exosomes encapsulate a diverse spectrum of miRNAs, including miR-19b, miR-20a, miR-126-3p, miR-130a-3p, miR-210-3p, and the longevimiR cluster miR-17-92. These miRNAs are implicated in crucial biological processes such as angiogenesis, cell cycle regulation, and hypoxic stress adaptation [94].

Exosomes derived from iPSCs exhibit a superior efficacy in cardiac tissue repair versus iPSCs themselves. While both enhance angiogenesis, curtail myocyte apoptosis, and bolster left ventricular (LV) function post-reperfusion following myocardial infarction, iPSC-derived exosomes are more effective with a lower risk of tumor formation, making them a safer alternative [94].

Another study assessed the efficacy of iPSC-derived exosomes for treatment of retina-associated disorders. Under hypoxic conditions, iPSC-derived exosomes reduced the frequency of apoptosis and increased proliferation in Müller cells, pivotal components of the retina that are vulnerable to hypoxic injury. This protective effect was attributed to the exosomes’ ability to deliver the Splicing factor proline- and glutamine-rich (SFPQ) protein to Müller cells, resulting in increased expression of histone deacetylase 1 (HDAC1); an increase in HDAC1 activity within Müller cells prompts the deacetylation and subsequent suppression of hypoxia inducible factor (HIF)-2α, a protein associated with retinal disorders and hypoxia-triggered damage. Elevated HIF-2α concentrations correlate with complications such as retinal neovascularization in ischemic retinal diseases induced by hypoxia [95].

### 3.5. Exosomes from Neural Stem Cells

Neural stem cells (NSCs) have significant potential as they can develop into neurons, astrocytes, and oligodendrocytes and thus have therapeutic promise in neurological diseases, such as neurodegenerative diseases and spinal cord injuries (SCI) [96].

NSCs are generally extracted from specific areas of the brain, notably the subventricular zone (SVZ) and the dentate gyrus in the hippocampus. Alternatively, they can be sourced from embryonic tissues or produced from iPSCs. Following sampling, enzymes are employed to dissolve the extracellular matrix, liberating the cells from the tissues. Thereafter, centrifugation segregates the stem cells based on density and size. The identity of NSCs can be verified using surface markers such as Nestin, SOX2, A2B5, and Neuron glia antigen-2 (NG2) [96], as illustrated in Figure 2.

NTA findings showed that most NSC-derived exosomes (NSC-exos) had a size distribution between 30 nm to 150 nm, peaking around 100 nm. TEM further confirmed their unique cup-shaped structure. The exosomes from NSCs prominently displayed surface markers like TSG101, CD9, and Flotillin-1 [97,98].

NSC-exos contain an abundance of miR-9. When introduced to neuronal cultures, miR-9 encouraged neurite growth and facilitated the transformation of neural progenitor cells into mature neurons. Although the precise mechanism has not yet been fully delineated, research indicates that miR-9 uptake by NSCs inhibits *Hes1*, a gene that typically keeps NSCs in an immature state, thus allowing NSCs to mature and differentiate. However, the study noted that this relationship is contingent on specific conditions and timing [97].

In addition, NSC-exos exhibited increased resilience under stressful conditions like oxidative stress or inflammation. These exosomes also supported the development of synaptic connections, playing a pivotal role in rejuvenating neural pathways in damaged tissues. Live studies on rodents with neural injuries showed that those treated with NSC-exos displayed noticeable improvements in recovery with improved motor skills and cognitive abilities compared to untreated animals, and tissue analysis morphologically corroborated neural tissue regeneration in the treated group [97].

Another investigation focusing on neuronal exosomes in the context of spinal cord injury revealed that these exosomes effectively inhibited the activation of astrocytes and microglia post-injury. Their activation is a primary factor in creating an environment that resists neuronal regeneration. Additionally, these exosomes encouraged the maturation of oligodendrocyte precursor cells (OPCs), neurite growth, and differentiation of neural stem cells, leading to enhanced neuronal and motor function at the injury site. While the exact exosomal component causing these effects remains unidentified, the exosomes were associated with decreased levels at the site of injury of complement C3, glial fibrillary acidic protein, CD80, CD163, and arginase 1, which help to reduce astrocyte and microglia activation. Conversely, increased levels of myelin basic protein, myelin-associated glycoprotein, and myelin oligodendrocyte glycoproteins were noted, factors that support the maturation of OPCs [98].

**Table 1:** Surface markers and therapeutic applications of exosomes from various sources.

Source of Exosome	Therapeutic Application	Surface Markers
Exosomes from Adipose-Derived Stem Cells	Tissue repair, specifically in bone fracture healing and limb ischemia. Boost angiogenesis in HUVECs.	CD9, CD63, and CD81 [76,77,78,79].
Exosomes from Bone Marrow-Derived Mesenchymal Stem Cells	Cardioprotection against doxorubicin-induced toxicity.	CD63, CD9, and TSG101 [86,87].
Exosomes from Hair Follicle-Derived Mesenchymal Stem Cells	Wound recovery and decreased oxidative stress in dermal fibroblasts.	CD13, CD9, CD63, CD105, CD81, CD29, CD44, CD49e; SSEA-4 [90,91].
Exosomes from Induced Pluripotent Stem Cells	Cardiac tissue repair. Treatment of retina-associated disorders.	CD9, TSG101; SSEA-1 [94,95].
Exosomes from Neural Stem Cells	Increased resilience under oxidative stress and inflammation and support development of synaptic connections.	TSG101, CD9, and Flotillin-1 [97,98].

## 4. Role of Exosomes in Diagnostics and Oncology

### 4.1. Enhancing Diagnostic Precision: The Emerging Role of Exosomes

Historically, a multitude of biomarkers have been developed and utilized as diagnostic instruments, even those possessing relatively low specificity [99]. The Prostate-Specific Antigen (PSA) test, a well-recognized diagnostic method employed for initial screening and determination of the need for transrectal ultrasound-guided prostate biopsy, exhibited a true-positive rate in merely 32.4% of biopsied individuals [100]. The remaining 67.6% were diagnosed with benign prostatic hyperplasia, which generally resolves without intervention [100].

Given this substantial rate of false-positive results, and the potential adverse effects of such invasive procedures [101], the scientific community continuously seeks precise non-invasive disease markers [102]. One study ascertained those exosomes expressing CD81 and PSA antigen on their surface demonstrated superior diagnostic value in prostate cancer detection compared to the free serum PSA antigen test [103]. This discovery could considerably enhance clinicians’ decision-making capabilities, facilitating the execution of transrectal ultrasound-guided prostate biopsies primarily in those patients who exhibit strong indications of the disease [104].

Since exosomes reflect the molecular properties of their source cells, they could yield invaluable insights for disease detection, surveillance, and the formulation of personalized treatment strategies [105]. While certain exosomes have demonstrated potential as standalone diagnostic markers, their incorporation alongside established tests, such as the PSA assay, could enhance their diagnostic accuracy and sensitivity [106]. For many years, traditional physical biopsy has been the gold standard for confirmatory testing for various diseases, including cancers. A physical biopsy can provide highly reliable results, but this is contingent upon obtaining a high-quality sample and necessitates physical access to the area of interest. This becomes challenging for relatively inaccessible regions, such as intracranial tumors [107].

In such cases, the current alternatives either rely upon imaging techniques, which often lack specificity for certain types of cancers [108], or necessitate surgery to excise the mass for analysis. Both approaches are sub-optimal, particularly given the advancements in medical technology, and it is here that liquid biopsies play a crucial role [109]. Since cells continuously release exosomes, they can serve as potential biomarkers with diagnostic value [110]. Exosomes offer a significant advantage over traditional tissue biopsies by providing a minimally invasive and easily repeatable strategy for evaluating disease status [110]. For example, serum exosomal-miR-122 was found to be as effective as tissue biopsy in diagnosing non-alcoholic fatty liver disease [111].

### 4.2. Impact of Cancer Stem Cell-Derived Exosomes on Cancer Aggressiveness and Diagnostic Applications

Cancer stem cells (CSCs) have emerged as pivotal in oncology, given that they contribute to cancer expansion and recurrence due to their ability to proliferate vigorously and exist in abundance. These cells, much like their non-cancerous counterparts, possess the unique ability to self-renew and differentiate, thereby driving tumor growth and heterogeneity. The niche, specialized microenvironment is crucial in regulating CSC behavior. Molecular intricacies within this niche environment can influence CSC proliferation, differentiation, and resistance mechanisms [112].

The niche environment is composed of various cellular and non-cellular components. These components interact in a complex manner to create a supportive microenvironment for CSCs. Amongst those, CSC-derived exosomes (CSC-exos) are emerging as a major player in the aggressiveness of cancer [113].

A study found that esophageal carcinoma stem cells (ECSCs) increased their self-renewal capacity by escaping immune surveillance, reporting that exosomes secreted by ECSCs contained a protein called O-GlcNAc transferase (OGT). This exosomal OGT was transferred to neighboring CD8+ T cells, increasing the expression of PD-1 in these immune cells. The programmed cell death protein (PD)-1/programmed death ligand-1 (PD-L1) pathway is an immunosuppressive mechanism debilitating the activity of cytotoxic CD8+ T cells. By increasing PD-1 expression, ECSCs increased their survival and proliferation [114].

In another study, lung cancer stem cells (LCSCs) enhanced the metastatic ability of surrounding non-stem cells of lung cancer. LCSCs release exosomes containing the bioactive miR-210-3p, which, upon transfer to other lung cancer cells, suppresses *FGFRL1* expression, thereby enhancing the metastatic traits of the recipient cells. Those exosomes increased the expression of N-cadherin, vimentin, matrix metalloproteinase (MMP)-9, and MMP-1, leading to increased lung cancer invasiveness and migratory potential [115]. Since CSC-exos play an important role in cancer aggressiveness, they can be used in the detection of cancer and in tracking cancer progression and resistance, which can help in predicting the prognosis of the disease. They could also help in tailoring the treatment plan for individual patients [116].

## 5. Potential Therapeutic Applications in Disease and Regenerative Medicine

### 5.1. Endocrine Disorders

#### 5.1.1. Diabetes Mellitus

**Figure 3:**
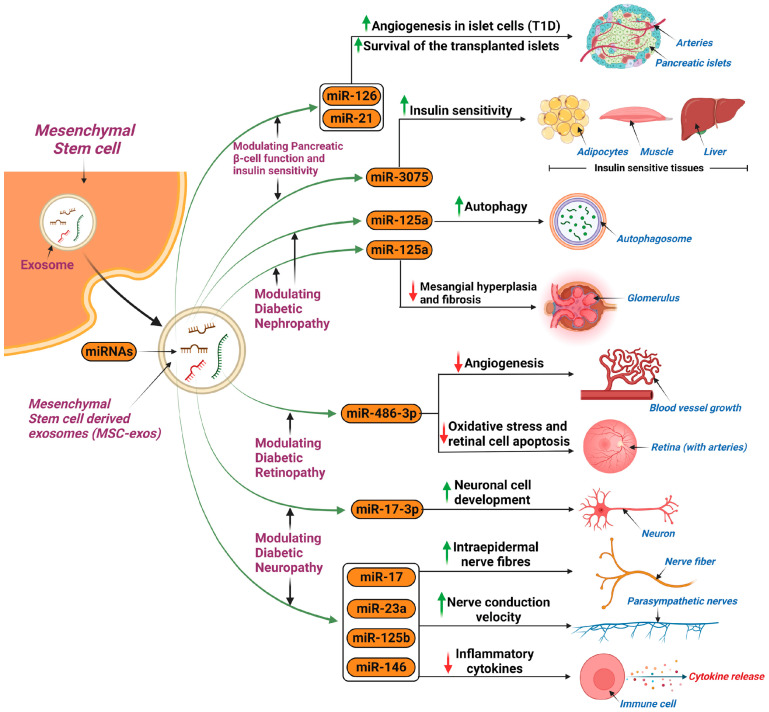
Therapeutic applications of MSC-exos in diabetes treatment and its microvascular complications. MSC-exos can be used to modulate a wide range of diabetes manifestations, including: pancreatic beta cell function and insulin sensitivity; diabetic retinopathy; diabetic nephropathy; and diabetic neuropathy. MSC-exos: mesenchymal stem cell-derived exosomes; T1D: type 1 diabetes mellitus; miR: microRNA. Upward green arrows indicate positive modulation and downward red arrows indicate negative modulation, respectively.

MSC-exos have been shown to limit disease progression in DM, particularly type 1 diabetes mellitus (T1DM) [117]. Specifically, MSC-exos have been found to harbor protective attributes that counteract the autoimmune destruction of pancreatic B islet cells; this is likely due to their ability to upregulate angiogenesis through increased VEGF expression (allowing revascularization of islets) and, in the realm of transplanted islet cells, they have been found to promote donor cell survival in hosts and also to increase the success rate of transplantation, as well as to promote the revascularization of the transplanted donor cells [118,119]. Molecularly, studies have shown that this occurs due to an increased ability to combat inflammatory cytokines by enhancing the function of regulatory T cells (Tregs) and inhibiting the proliferation of peripheral blood mononuclear cells (PBMCs), all of which occurs through the exosomes ability to deliver miRNA [120,121]. Furthermore, MSC-exos which trigger the AMP-activated protein kinase (AMPK) pathway can directly enhance glucose metabolism and are rich in miR-3075, which can promote insulin sensitivity, hence further demonstrating therapeutic efficacy in diabetes [122]. However, it is important to note the benefits of MSC-exos are dependent on their cargo, as exosomes containing other variants of RNA can produce the opposite effects. For example, MSC-exos rich in miR-210 limit glucose uptake, and exosomes with reduced levels of insulin receptor substrate 1 and hormone sensitive lipase can promote insulin resistance (IR) [122]. Figure 3 summarizes the applications of MSC-exos in the treatment of diabetes mellitus and its microvascular complications.

##### Diabetic Nephropathy

A study performed on a streptozotocin-induced diabetic rat model found clinical potential for the use of MSC-exos in diabetic nephropathy. Autophagy was induced amongst the experimental group, as was confirmed by the increased expression of autophagy markers (LC3, Beclin-1), as well as decreased mTOR activity and reduced expression of fibrotic markers, all of which demonstrate the effectiveness of MSC-exos in attenuating diabetic nephropathy [123]. MiR-125b can promote this process [124]. The novel effects of MSC-exos were also further highlighted in another study using a diabetic rat model, where MSC-exos carrying miR-125a decreased mesangium hyperplasia and fibrosis, likely via the inhibition of HDAC1 and endothelin 1 (ET-1) [125].

##### Diabetic Retinopathy

MSC-exos were found useful for preventing the progression of diabetic retinopathy (DR). In particular, BMSC-exos and human umbilical cord (HUCMSC-exos) proved to be useful [126]. The former was found to inhibit angiogenesis when loaded with miR-486-3p, as well as oxidative stress, and retinal cell apoptosis, via a combination of interactions involving the downregulation of the Toll-like receptor (TLR)4/Nuclear factor kappa-light-chain-enhancer of activated B cells (NF-κB) pathway, and the miR-34a-5p/X-box binding protein 1 (XBP1) pathway, whereas the latter is more involved with promoting the development of neuronal cells, whilst also inhibiting its apoptosis, via the activation of the brain-derived neurotrophic factor (BDNF)-Tropomyosin receptor kinase B (TrkB) signaling pathway and the targeting of *STAT1* [127,128].

##### Diabetic Peripheral Neuropathy

In a study using diabetic mouse models, MSC-exos produced a substantial increase in nerve conduction velocity, and the mice were found to have an increased number of intraepidermal nerve fibers. Moreover, the mice were found to have reduced levels of inflammatory cytokines, attributable to miRNAs that inhibited the TLR4/NF-κB signaling pathway, namely miR-17, miR-23a, and miR-125b, thereby mitigating neuronal dysfunction [129]. A comparable study, again using diabetic mouse models, used MSC-exos loaded with miR-146a, and concluded there was potential to improve clinical outcomes in patients suffering from diabetic peripheral neuropathy via the above mentioned pathway [130].

#### 5.1.2. Polycystic Ovary Syndrome

SC-exos have demonstrated a wide range of capabilities in managing polycystic ovary syndrome (PCOS) and its complications. Specifically, ADSC-exos were able to enhance fertility and protect against the metabolic complications in rat models, particularly through the miR-21-5p-loaded exosomes which were found to enhance the hepatic metabolism by activating the insulin receptor substrate 1 (IRS1)/Akt pathway [131]. This alteration of hepatic metabolism is ultimately due to reduced btg2 transcription activity, thus promoting glucose uptake and reducing the degree of steatosis and fatty vacuoles [132]. Subsequently, improved metabolic outcomes and fertility were displayed. Moreover, ADSC-exos also pose a structural benefit in PCOS, where there was a reduction in the number of cystic follicles and an increase in the number of corpora lutea and oocytes [131].

### 5.2. Gastrointestinal Disorders (Inflammatory Bowel Disease)

Inflammatory bowel disease (IBD) is a growing health burden, and hence greater research initiatives are being placed towards its treatment, including the use of sc-exos. BMSCs are of particular importance as they can strengthen the mucosal barrier by polarizing M2b macrophages leading to reduced inflammation whilst also preventing the development of fibrosis. The cargo of these exosomes contain proteins with various anti-colitis properties, especially metallothionein-2 [133,134]. Further relevant cargo include various miRNAs, which play a crucial role in immune system coordination through regulating T cells and NF-κB pathways; these miRNAs include miR-146a and miR-155 [135]. Furthermore, it has also been demonstrated that exosome treatment in IBD can normalize the intestinal microbiome, especially via IPSC-derived exosomes [136]. The same study also noted increased proliferation of intestinal epithelial cells, increased leucine-rich repeat-containing G protein-coupled receptor 5 (Lgr5) intestinal stem cells, and further intestinal angiogenesis, through ADSC-exos and IPSC-derived exosomes. More recently, HUCMSC-exos have also been shown to play a role in IBD treatment, as demonstrated in mouse models, particularly through their alleviation of intestinal fibrosis, which is brought on as a late sequalae of IBD. This was primarily achieved via inhibiting the phosphorylation of the extracellular-signal-regulated kinase (ERK) pathway, which led to a decrease in fibrotic factors, such as fibronectin and collagen I [137].

### 5.3. Cardiovascular Disease (Myocardial Infarction)

A growing body of research strongly supports the use of MSC-exos in the setting of diseased cardiac tissue [138,139]. Numerous studies have found that MSC-exos from various sources have the ability to preserve cardiac tissue when under the stress of ischemia or reperfusion; one study found that MSC-exos loaded with miR-125b-5b exerted an anti-apoptotic effect on cardiomyocytes via the suppression of *p53* and BCL2 antagonist/killer 1 (*BAK1*) genes [140]. Another study confirmed the anti-apoptotic effects, further finding that MSC-exos loaded with miR-22 targeted the methyl CpG binding protein-2 (*MECP2*) gene which promotes cardiac preservation and reduces fibrosis [141].

Increased angiogenesis is also another mechanism whereby MSC-exos were found to be beneficial in cardiac infarcts. Numerous studies using mouse models have found that one crucial underlying cause is the increased proliferation of HUVECs which, in turn, contributes to tube formation and angiogenesis [142,143]. One mechanism for this upregulated proliferation of HUVECs is increased expression of the *RASA1* gene (via miR-132), and transplanted miR-132 exosomes into ischemic cardiac tissue was reported to result in neovascularization of the peri-infarct zones whilst maintaining cardiac function [142]. Interestingly, another method of promoting angiogenesis is through the increased secretion of platelet-derived growth factor (PDGF)-D from umbilical cord MSC-exos overexpressing protein kinase B [144]. As opposed to the HUVEC proliferation mechanism, one study found that adipocyte MSC-exos have a profound effect on endothelial progenitor cells (EPCs) in patients suffering acute myocardial infarction, as exosomes overexpressing sirtuin-1 (SIRT1) caused increased expressions of CXC chemokine-12 (CXCL12) and nuclear factor E2 related factor 2 (Nrf2) in those EPCs. This resulted in a reduction in infarct size, reduced ventricular remodeling, and enhanced angiogenesis [145]. The ability to suppress inflammation is another feature of MSC-exos in the setting of myocardial infarction. This occurs in a manner similar to that discussed in DM, via the promotion of Treg cells (through c-Fos targeting) and the inhibition of PBMC proliferation. This effect was profound in MSC-exos overexpressing miR-181a [146].

### 5.4. Cancer

The use of MSC-exos in cancer has broad applications and research continues to develop novel uses for these vesicles. MSC-exos have the ability to enhance drug sensitivity of malignant cells to chemotherapeutic agents [147]. This property has been particularly useful in hepatocellular carcinoma (HCC), where an in vivo study found that the use of ADSC-exos loaded with miR-122 via intratumor injection enhanced the malignant cell sensitivity to sorafenib; yet, it is important to note that the MSC-exos themselves did not impact tumor growth [148]. Another study found that ADSC-exos loaded with miR-199 were able to target and inhibit the mTOR pathway in HCC, which enhances tumor sensitivity to doxorubicin [149]. It is the ability of exosomes to deliver their cargo that produces these advantageous results. Gliomas have also been targeted for MSC-exo therapy, with the overexpression of miR-199a having a profound effect on tumor sensitivity to temozolomide with inhibition of tumor growth, likely due to a downregulation of ADP-ribosylation factor GTPase-activating proteins with a GTPase domain, ankyrin repeat, and PH domain 2 (AGAP2) [150].

Increasingly, studies have focused on MSC-exos as a potential drug delivery system in cancer therapeutics [151]. MSC-exos are particularly useful because of their high penetrative ability, long half-life, small size, and that they do not provoke an immune response [152]. This vehicular function is a relatively new area of research; however, an earlier study focused upon their use in pancreatic cancer in murine models, where MSCs were primed with paclitaxel, a chemotherapeutic agent, and the resultant MSC-exos were able to deliver the agent, suppressing tumor proliferation with a high degree of cell-specificity [153]. Another study found advantageous vehicular effects on melanomas in mouse models [154]; MSC-exos containing tumor necrosis factor-related apoptosis-inducing ligand (TRAIL) were injected into the tumor, and demonstrated anti-tumor activity with a delayed appearance of tumor, though this did require multiple injections of the MSC-exos [154]. The significance of this functionality was also seen in triple-negative breast cancer, where one study found that MSC-exos coated with specific integrins (modified disintegrin and metalloproteinase 15) on the membrane were able to co-deliver doxorubicin and cholesterol-modified miR-159 and demonstrated, both in vivo and in vitro, significant synergistic antitumor effects [155]. Overall, research has provided a solid basis for the use of MSC-exos as drug transportation vehicles.

**Figure 4:**
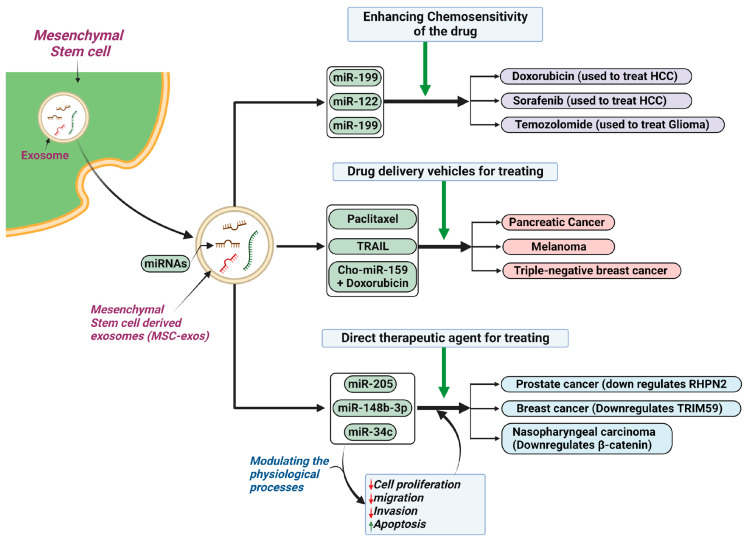
Therapeutic applications of MSC-exos in cancer treatment. The applications of MSC-exos for cancer can be broadly divided into 3 categories: to enhance chemosensitivity, to deliver drugs, and as a direct therapeutic agent via its miRNA cargo. Upward green arrow indicate positive modulation. Downward red arrows indicate negative modulation. MSC-exos: mesenchymal stem cell-derived exosomes; miR: micro-RNA; HCC: hepatocellular carcinoma; Cho: cholesterol-modified; RHPN2: rhophilin Rho GTPase binding protein 2; TRIM59: tripartite motif containing 59. Green arrows indicate the modulation of the respective pathways.

MSC-exos can also function as a direct therapeutic agent against malignant cells, primarily attributable to the miRNA they contain [156]. A study demonstrated this in breast cancer, where HUCMSC-exos containing miR-148b-3p minimized gene expression of tripartite motif 59 (TRIM59), thereby inhibiting cell proliferation and migration and promoting apoptosis, both in vitro and in vivo [157]. Similar results were seen in prostate cancer, where BMSC-exos carrying miR-205 targeted and suppressed the rhophilin Rho GTPase-binding protein 2 (*RHPN2*) gene, thereby suppressing proliferation, migration, and invasion whilst increasing apoptosis [158]. A further study on nasopharyngeal carcinomas reported that the expression of β-catenin decreases as a result of increasing levels of miR-34c delivered by MSC-exos with a reduction in cell proliferation, invasion, and migration and, interestingly, an enhanced radiosensitivity resulting in apoptosis [159]. Figure 4 summarizes the current therapeutic applications of MSC-exos for cancer treatment.

### 5.5. Plastic Surgery

The skin functions as the primary immunological barrier and plays a crucial role within the immune system framework [160]. Cutaneous injuries, prevalent across various body regions, represent the most common wound [161]. These injuries pose a significant healthcare challenge, exerting substantial economic pressure on both individuals and healthcare infrastructures [162]. Particularly, the management of surgical wounds and the treatment of diabetic ulcers are associated with the highest financial burdens related to wound care [163]. Recent studies have demonstrated the effectiveness of MSC-exos in promoting wound healing, representing a critical milestone in the field of regenerative medicine. Exosomes influence the essential phases of wound healing, which are governed by various signaling pathways [161]. These phases include hemostasis, inflammation, angiogenesis, proliferation, and remodeling [161].

Exosomes have been identified as playing a significant role in mitigating inflammation during the inflammatory phases. They achieve this by inhibiting the proliferation of blood mononuclear cells and facilitating the transformation of regulatory T cells which, in turn, reduces lymphocytic infiltration in the skin [164]. Furthermore, this process involves the downregulation of inflammatory cytokines, along with decreases in the numbers of mast cells, eosinophils, and in IgE levels, highlighting a targeted approach towards reducing inflammation [161,165].

As the healing process enters the angiogenic stage, specialized exosomes, treated with deferoxamine and atorvastatin, demonstrate their potential as therapeutic agents by enhancing angiogenesis in wounds, specifically in cases of diabetic ulcers. This enhancement is achieved through the activation of pathways such as PTEN/PI3K/Akt and Akt/Endothelial nitric oxide synthase (eNOS), facilitating the formation of new blood vessels and contributing to the overall healing process [161,166,167]. Similarly, MSC-exos can promote the migration and proliferation of vital cell types such as keratinocytes and fibroblasts during the proliferative stage [167]. This promotion is achieved through the inhibition of specific signaling pathways, including Large tumor suppressor kinase 2 (LATS2) [168], Peroxisome proliferator-activated receptor γ (PPARγ) [169], and Apoptosis-inducing factor (AIF) nucleus translocation [170], while concurrently activating others, such as PI3K/Akt [171], Akt/Hypoxia-Inducible Factor 1 alpha (HIF-1α) [171,172], ERK1/2 [173], and Wnt/β-catenin [174].

In the final stage of wound healing, remodeling, MSC-exo therapy presents another opportunity to influence the healing process [161]. At this stage, MSC-exos assist in the transition from granulation tissue to the formation of more permanent scar tissue, a critical phase where abnormal healing, such as keloids and hypertrophic scars, may occur [161]. By modulating the TGF-β/Smad2 pathway [175,176], MSC-exos can inhibit the fibroblast–myofibroblast transition and regulate collagen synthesis, thereby diminishing scar formation and enhancing the aesthetic results of wound healing [177].

### 5.6. Neurodegenerative Disorders

A key focus of new sc-exos research is to combat neurodegenerative disorders. One example is multiple sclerosis, where Clark et al. have found therapeutic potential in using placental MSC-exos (PMSC-exos) with non-specific cargo and discovered a role in myelin regeneration in experimental autoimmune encephalitis-induced murine models (mimics multiple sclerosis), as well as reduced DNA damage to oligodendrocytes [178]. Moreover, another study has found potent effects on neurobehavioral symptoms, as well as the effects of limited disease progression and inflammation; this was achieved through BMSC-exos (with a non-specific protein cargo) targeting and polarizing the microglia from M1 (pro-inflammatory variant) to M2 (neuroprotective variant) [179]. Moreover, limiting multiple sclerosis progression was also seen by the targeting of lymphocytes by limiting their activation, proliferation, and secretion of inflammatory agents using BMSC-exos loaded with Programmed Cell Death Ligand 1 (PD-L1), TGF-β, and galectin (GAL)-1 [180,181].

In Alzheimer’s disease, it was found that MSC-exo therapy can improve brain glucose metabolism and cognitive function in mouse models, overall contributing to a slower rate of disease progression [182]. Furthermore, one study demonstrated enhanced neurogenesis in the subventricular zone (a site responsible for the production of glia and neurons) and beta amyloid-induced cognitive dysfunction. These effects were comparable to the use of MSCs whilst mitigating the previously mentioned risks of stem cells [183]. The mechanism of reduced beta amyloid-induced neurotoxicity is thought to be due to inhibition of the AKT/Glycogen synthase kinase-3 beta (GSK-3β)/β-catenin pathway, which is responsible for mediating growth differentiation factor-15 [184].

Promising results have also been shown for Parkinson’s disease, from both a diagnostic and therapeutic perspective. The former is related to various expression patterns of proteins in the Parkinson’s disease exosomes. For example, multiple studies demonstrated exosomal α-synuclein to be elevated in Parkinson’s disease subjects compared to controls [185,186,187]. Furthermore, one study found diagnostic potential in distinguishing the types of Parkinson’s disease using exosomal α-synuclein (tremor dominant vs. non-tremor dominant), where the tremor-dominant group expressed significantly higher levels of α-synuclein [187,188]. Therapeutically, one study found that exosomes of stem cells from the dental pulp of human exfoliated deciduous teeth reduced dopaminergic neuronal cell apoptosis by approximately 80% when grown on laminin-coated three-dimensional alginate micro-carriers, but the effects were not reproduced under standard culture conditions [189]. Moreover, MSC-exos can also promote angiogenesis in human brain microvascular endothelial cells by increasing the expression of *ICAM1*, with subsequent downstream activation of *SMAD3* and *P38MAPK*, which shows promising therapeutic potential for the treatment of Parkinson’s disease [190]. Although still in the early phase of research, the opportunity for future applications of sc-exo therapy in Parkinson’s disease appears hopeful.

### 5.7. Lung Diseases

MSC-exos offer a promising therapeutic approach for managing various lung diseases through multifaceted mechanisms. These exosomes, miRNAs, navigate through complex cellular pathways to exert anti-inflammatory, tissue reparative, antifibrotic, and immunomodulatory effects, addressing the pathological hallmarks of lung disorders such as chronic obstructive pulmonary disease (COPD), acute lung injury (ALI), and pulmonary fibrosis [191].

The anti-inflammatory action of MSC-exos is crucial, especially in conditions like COPD and ALI, where the inflammatory cascade plays a significant role in disease progression. MSC-exos contain miRNAs, such as miR-451 and miR-27a-3p, that target the TLR4/NF-κB signaling pathway. By downregulating this pathway, MSC-exos reduce pro-inflammatory cytokine production, mitigating inflammation and protecting lung tissue from further damage. For instance, miR-451 modulates macrophage polarization towards the M2 phenotype, promoting tissue repair and resolving inflammation, which is crucial for attenuating conditions like burn-induced ALI [191].

Beyond their anti-inflammatory effects, MSC-exos facilitate lung tissue repair by enhancing cell proliferation, migration, and angiogenesis. This is partly achieved through the modulation of autophagy via targeting the mTOR signaling pathway with miRNAs such as miR-100 and miR-377-3p [191,192].

The antifibrotic actions of MSC-exos are particularly relevant in combating pulmonary fibrosis, characterized by excessive extracellular matrix deposition and impaired lung function [193]. MSC-exo-derived miRNAs, including miR-29b-3p and miR-186, target fibrosis-related pathways such as the frizzled 6 (FZD6) and SRY-related HMG-box 4 (SOX4)/dickkopf 1 (DKK1) pathways, respectively [193]. By inhibiting fibroblast activation and collagen synthesis, these miRNAs prevent the progression of fibrotic changes in the lung, showcasing the antifibrotic potential of MSC-exos [191].

In the context of addressing the urgent need for treatment options for Acute Respiratory Distress Syndrome (ARDS), research has pointed towards the promising safety and efficacy of EXO-CD24 in over 180 ARDS patients across phase 1b/2a and phase 2b studies. EXO-CD24 utilizes the immunomodulatory capabilities of CD24-enriched exosomes. CD24, a heavily glycosylated, membrane-anchored protein, plays a critical role in immune regulation by differentiating Damage-Associated Molecular Patterns (DAMPs) from Pathogen-Associated Molecular Patterns (PAMPs), thus facilitating immune discrimination between self and non-self entities. By binding to DAMPs, CD24 prevents their interaction with pattern recognition receptors (PRRs) like TLRs and NOD-like receptors (NLRs), effectively inhibiting the activation of the NF-ĸB pathway, a key mediator of inflammatory responses. This action contrasts sharply with other anti-inflammatory treatments that either target specific cytokines or broadly suppress the immune system through steroids. EXO-CD24’s mechanism operates upstream, reverting the immune system to its normal activity without compromising pathogen defense [194,195].

## 6. Overcoming Challenges of Working with Exosomes in the Laboratory

### 6.1. Current Methods for Exosome Isolation

Exploring the methodologies for isolating exosomes is of utmost importance due to their small size (30–100 nm), low density (1.13–1.19 g/mL), and the presence of similar constituents (cell fragments, proteins) in bodily fluids. These factors pose significant challenges in achieving successful separation [196]. Several commonly employed separation methods include ultrafiltration, ultracentrifugation, chromatography, and immunoaffinity, the features of which are compared and contrasted in Table 2. Understanding and comparing these methods will contribute to the advancement of exosome research, thereby aiding their use in clinical settings.

### 6.2. Standardization of Isolation

As there are a variety of exosome isolation protocols, and exosome heterogeneity and complexity is apparent, standardization is necessary to minimize pre-isolation and analytical variables. Using serum-free media is suggested as a growth supplement for cell culture. Additionally, it is recommended that a needle with a gauge that does not induce shear forces is used to avoid inducing platelet aggregates downstream during blood collection. Another important standardization parameter is the timing and fasting/fed state of the blood collection as this impacts the content of the exosomes. Characteristics of the recipient are also important, such as age, gender, race, comorbidities and related treatments [202].

### 6.3. Current Methods for Exosome Characterization

Once exosomes are successfully isolated from the sample, they must undergo a characterization process to determine their size, morphology and, potentially, their content. Numerous methods are emerging in this field, with NTA, transmission electron microscopy (TEM), and scanning electron microscopy (SEM) being prominent techniques. NTA provides valuable information about size and concentration, while TEM offers insights into the exosome shape. Depending on the research focus, additional analyses can be employed, such as dynamic light scattering (DLS), liquid chromatography with tandem mass spectroscopy (LC-MS-MS), two-dimensional gel electrophoresis (2DGE), reverse transcriptase quantitative polymerase chain reaction (RT-qPCR), and fluorescent microscopy (FM) [203].

### 6.4. Risk Considerations

Exosomes play a role in transporting components to recipient cells, although the specific mechanisms of their internalization and accumulation into target cells are still not fully understood. One hypothesis, known as the “the next cell hypothesis”, suggests that exosomes facilitate communication between neighboring vesicles, which can contribute to the development of diseases like diabetes or the progression of Alzheimer’s disease [204].

Another risk is that when exosomes are extracted from human B lymphocytes, there is induction of antigen-specific major histocompatibility complex T cell responses. Further, promotion of oncogenes via transfer of oncogenic miRNAs among cancer cells and tumor stroma is also evident. Moreover, inflammatory responses can occur in the recipient cell due to the presence of exosomes [205].

## 7. Exosome Research: From Bench to Bedside and Future Perspectives

### 7.1. Current Clinical Trials

Through preclinical studies and clinical trials, exosomes have demonstrated promise in the treatment of diseases (Table 3), specifically their regenerative effects in the field of oncology [206]. MSC-exos have great potential for clinical applications due to their nanoscale size as they can be accurately delivered to the site of interest with minimal risk of aggregate formation. Exosomes can be isolated from immortalized MSCs and used clinically in ways that MSC immortalized cells cannot. There are several ongoing preclinical studies in animal models that showcase the promising therapeutic effect of MSC-derived exosomes for the treatment and prevention of diseases such as neurological disorders and autoimmune diseases, in addition to their cardiac, renal and hepatic regenerative properties [207].

One published clinical trial utilized MSC-exo therapy in patients with chronic kidney disease (CKD). End stage renal disease is caused by the irreversible decline of renal function, the main characteristic being tubulointerstitial fibrosis. Nassar et al. conducted the first randomized controlled clinical trial in Egypt using HUCMSC-exos in stage 3 and 4 CKD patients to slow disease progression [208]. The treatment group received a weekly dose of 100 mcg/kg of body weight for two weeks whilst the control group received a placebo. Administration of the doses were intravenously and intra-arterially in the first and second doses, respectively. The results showed improvement in estimated glomerular filtration rate, and other renal function parameters such as blood creatinine and urea levels. Another published trial using MSC-exos to successfully treat skin hyperpigmentation is included in this review [209]. A randomized placebo-controlled study showcased the therapeutic effect of exosomes extracted from adipose tissue on skin hyperpigmentation. One group received 0.2 g of AMSC-exos while the other group received 0.2 g of placebo. Results showed short term improvement in hyperpigmentation in the treatment group through reduction in melanin However, it was followed with relapses [207].

Preliminary results from an ongoing trial by Zali et al. [210] has indicated the impact of BMSC-exos in patients with acute ischemic stroke (AIS). AIS participants were given miR-124-transfected MSC-exos in the ischemic region of the brain. Evaluated parameters included side effects such as recurrent stroke, seizures and oedema development. Potential efficacy was measured by improvement based on the manipulated Rankin Scale one-year post-treatment and measurement of AIS patient disability (with a score ranging from 0 to 6). An ongoing trial led by Wang et al. [211,212] aims to evaluate the efficacy of MSC-exos in mild to moderate Alzheimer’s dementia. The participants are being divided into 3 groups based on dosage, with one group receiving a low dose (5 mcg) of exosomes derived from adipose tissue through a nasal drip twice a week, the second group received a higher dose (10 mcg) of exosomes twice a week, and the third group receiving the highest dose (20 mcg). Kidney and liver function will be monitored and cognitive subscales will be utilized to determine cognitive scale and functionality. The role of exosomes in the treatment of COVID-19 was led by Shanghai Public Health Clinical Center and Wihan Jinyintan Hospital in an ongoing clinical trial at Wuhan, China where 24 PCR-confirmed COVID-19 patients were given 5 inhalation doses of MSC-exos for 5 days. Safety follow-up involves adverse event and severe adverse event parameters initially after treatment for 28 days and the time for clinical improvement [207,213].

**Table 3:** Clinical Trials for Exosomes of Various Stem Cell Sources.

Condition Treated	Phase	Patient Group	Type of MSC-Exos	Dosage	Administration Method	Main Outcomes	Trial Reference
CKD Stage 3 and 4	Phase II/III	Stage 3 and 4 CKD patients	HUCMSC-Exos	100 mcg/kg weekly for two weeks	Intravenous and intra-arterial	Improvement in glomerular filtration rate, reduction in blood creatinine and urea levels	[208]
Skin hyperpigmentation	Not specified	Individuals with hyperpigmentation	ADSC-Exos	0.2 g of ADSC-exos twice a day for 8 weeks	Local	Short-term improvement in hyperpigmentation through reduction in melanin; however, followed by relapses	[207]
AIS	Phase I/II	AIS patients	miR-124-transfected MSC-Exos	Not specified	Intraparenchymal or stereotaxis	Incidence of treatment-emergent adverse events and improvement based on the modified Rankin Scale; evaluation of AIS patient disability.	[210]
Mild-to-moderate Alzheimer’s, dementia	Phase I/II	Patients with mild-to-moderate Alzheimer’s based on NIA/Aa	Adipose Tissue-Derived Exosomes	Low dose (5 mcg); Medium dose (10 mcg); High dose (20 mcg) twice a week	Nasal drip	Number of participants with abnormal laboratory values and adverse events by CTCAE v4.0 at 12 weeks. Cognitive function, quality of life evaluation, MRI neuroimaging, PET-CT neuroimaging, and changes in AD biomarkers are secondary outcomes.	[211,212]
COVID-19	Phase I	PCR-confirmed COVID-19 patients	MSC-exos	5 inhalation doses over 5 days	Inhalation	Adverse reaction and severe adverse reaction up to 28 days.	[207,213]

### 7.2. Challenges and Limitations of Exosomes

The maintenance of EVs continues to be a challenge, as it has been shown that long-term storage of these vesicles is most stable at temperatures around −80 °C, which also poses a secondary challenge, as such low temperatures can minimize translational activity [214,215,216,217]. One method to tackle this is freeze-drying exosomes with the removal of water from a frozen specimen under a vacuum, and this can minimize the strict storage requirements of exosomes and prolong their shelf life [218]. However, an additional challenge arises with freeze-drying, in that the aggregation of the EVs can consequently lower their efficacy to act on target sites; however, stabilizers, such as glucose or starch, can be added to minimize the clustering in the freeze-drying process [216].

Another challenge that can arise is sterility [219]. Various viruses and exosomes are similar in size and, hence, viral material can contaminate exosomes [220]. This is particularly true in the biogenesis phase, where it was shown that retroviruses such as the human immunodeficiency virus-1 and the human T-lymphotropic virus type 1 can use exosomes as biocarriers and later spread throughout the body whilst evading the immune response [221]. To mitigate this risk, there should be careful monitoring of any pathogens prior to application.

The lack of standardized isolation protocols also poses a challenge. Given the recent emergence of the field of SC-exos, there is no set protocol to isolate these vesicles, hence leading to pleiotropic effects given the variations in quality and quantity [219]. This also increases the risk of contamination and creates safety concerns, with the further risk of damaging the exosomes [219]. Future research must be conducted to optimize the isolation process and to create safe and standardized methodologies to further purify exosomes.

### 7.3. Emerging Areas of Research and Potential Breakthroughs

Emerging platforms are offering alternatives to traditional methods that depend on exosomes’ physical and chemical properties. Microfluidics and asymmetric flow field flow fractionation (AF4) stand out due to their sensitivity, time-efficiency, high recovery rate, and minimal volume requirements, making them promising for future personalized medicine [222].

#### 7.3.1. Microfluidics

Microfluidics encompass both active and passive methods that optimize recovery rates, isolation capacity, and input samples. These techniques include acoustophoresis, filtration, and viscoelastic flow to increase isolation efficiency. Isolation of exosomes based on density involves a label-free extraction of exosomes through the coupling of microfluidic designs with nanoparticles through centrifugal forces. Microhydrodynamics with centrifugation utilizes U-CENSE, a microchip that forces samples into subcategorized microchannels (serpentine inlet channel, microfluidic separation channel, and two outlets). This is advantageous as it enables a rapid detection of exosomes, and hence does not require external elements such as syringe pumps. As opposed to conventional ultracentrifugation, exosomes and particles in separation channels will encounter various forces including centrifugal, coriolis, buoyancy, and hydrodynamic drag. Acoustophoresis relies on mechanisms that operate based on size. Isolated targeted molecules are extracted based on the exertion of acoustic waves through two standard techniques, which are bulk acoustic waves that generate at specific frequencies and vibrate the transducer as a bulk. By contrast, surface acoustic waves initiate an acoustic pressure field through voltage on interdigital transducers, causing displacement with a range of frequencies that are biocompatible, not altering its cellular properties. The main limitations include the challenging and time-consuming technical procedures and the need for alignment precision. The technique is affected by factors such as density and compressibility, as well as particle size. Filtration in microfluidics is also based on size, where nano-porous membranes and filters are utilized in microchannels in microfluidics platforms to deliver advanced filtration methods. Woo et al. have developed the exodisc, a device that allows for label-free exosome purification through nanofiltration and centrifugation with spinning at a low rate through different pore sizes ranging from 600 to 30 nm [223]. This device, unlike standard ultracentrifugation methods, has a total recovery rate of 95% within a time span of 30 min. Another passive method introduced by Villarroya-Beltri et al. [31] traps exosomes using ciliated micropillars versus cells that are too large to enter the micro-pillared regions. Drawbacks include a lack of specificity, as particles that are of similar size to exosomes can be trapped. In addition, the process can induce cell lysis and debris accumulation on the nanowires, causing device clogging and thereby impeding the capture of target molecules [222].

In microfluidics, isolation based on functionality involves microchannel surface antibody modification and the utility of affinity particles and magnetic beads. He et al. have integrated magnetic beads with antigens for exosome capture/isolation, allowing for the manipulation of exosomes through external magnetic fields [204]. To specifically detect protein biomarkers, antibody-labeled magnetic beads were used to capture exosomes. Plasma samples were then combined with a lysis buffer to release intra-vesicular proteins from the captured exosomes for subsequent analysis. The major challenge is the high dependency on specific antibodies for each target of interest, in addition to device clogging [222].

#### 7.3.2. Asymmetric Flow Field Fractionation

AF4 is a separation method that uses a thin laminar flow film and does not rely on a stationary phase. This film is subjected to a force field at a perpendicular angle. Recently, AF4 has emerged as a valuable technique for separating exosomes. Especially when paired with other molecular assay methods, it proves highly effective for the fractionation of extracellular vesicles [202].

## 8. Conclusions

In conclusion, the therapeutic applications of sc-exos continue to grow in the field of regenerative medicine, where sc-exo-based therapies are being considered for a range of organ pathologies. Despite the similarities between sc-exos and SCs, the shift in research focus to the former is likely attributable to its cell-free features: lower tumorigenicity and lower immunogenicity. Moreover, the outcomes of sc-exo therapies are dependent upon their cargo and, hence, by altering the cargo, a more individualized therapeutic effect can be achieved, which is ideal for stem cell-based personalized medicine. As with any relatively new field, challenges remain and research is needed to better understand the mechanisms and applications of sc-exo therapies, to continue to monitor for adverse events, and to persist with refining and improving isolation and characterization techniques.

Moving forward, the future insights into sc-exos in regenerative medicine appear promising. Growing research efforts will likely discover newer therapeutic applications and refine current techniques, which can allow us to embrace widespread clinical adoption. Moreover, the integration of sc-exos and regenerative medicine can enhance precision medicine and bring individualized treatment to a range of medical conditions. Further research is needed to assess the application of this novel modality in a human population.

## Figures and Tables

**Table 2:** Features of various separation methods for exosome isolation.

Method	Description	Advantages	Limitations
Ultrafiltration	Separates based on size/weight [197].	Easy with no need for special equipment.	Time -consuming, and may not be suitable for blood samples, and clogging of filtration membrane.
Chromatography	Uses molecular size for separation [198].	Gentle isolation, preserves exosome integrity.	Limited from blood (1–5%) [199].
Ultracentrifugation	Relies on density/size differences [200].	Effective for large samples.	Labor-intensive, potential damage to exosomes [201].
Immunoaffinity	Targets specific proteins for isolation [197].	High specificity.	Requires specific antibodies and validation, which is expensive. Requires concentrated small volumes [197].

## Abbreviations

SC	stem cells
MSCs	mesenchymal stem cells
Sc-exos	stem cell-derived exosomes
EVs	extracellular vesicles
CSF	cerebrospinal fluid
DM	diabetes mellitus
hESCs	human embryonic stem cells
iPSCs	induced pluripotent stem cells
TGF-ß	transforming growth factor beta
VEGF	vascular endothelial growth factor
IL	interleukin
CCL7	chemokine ligand 7
CxCL2	chemokine ligand 2
miRNA, miR	microRNA
ncRNA	non-coding RNA
ESE	endosomes
CME	clathrin-mediated endocytosis
ClE	clathrin-independent endocytosis
ARF6	ADP-ribosylation factor 6
CLIC	clathrin-independent carriers
GEEC	glycosylphosphatidylinositol-enriched endocytic compartment
LSE	late-sorting endosome
GDP	guanosine diphosphate
ILVS	intraluminal vesicles
MVBs	multivesicular bodies
ESCRT	endosomal sorting complex required for transport
S1P	sphingosine 1 phosphate
EGFR	epidermal growth factor receptor
mRNA	messenger RNAs
CRC	colorectal cancer
CEA	carcinoembryonic antigen
CA19-9	cancer antigen 19-9
sEV	small EVs
NSCLC	non-small-cell lung cancer
NSCLC-sEVs	NSCLC-derived sEVs
SCLC	smallcell lung cancer
GPI	glycosylphosphatidylinositol
PI3K	phosphatidylinositol-3-kinase
PLC	phospholipase C
ADSCs	Adipose-derived stem cells
TEM	Transmission Electron Microscopy
ADSC-exos	ADSC-derived exosomes
ACER2	alkaline ceramidase 2
HUVECs	human umbilical vein endothelial cells
BMSCs	bone marrow-derived stem cells
NTA	nanoparticle tracking analysis
BMSC-exos	BMSC-derived exosomes
HFMSCs	Hair follicle-derived mesenchymal stem cells
HFMSC-exos	HFMSC-derived exosomes
TFs	transcription factors
LV	left ventricular
HDAC1	histone deacetylase 1
HIF	hypoxia inducible factor
NSCs	neural stem cells
SVZ	subventricular zone
NSC-exos	NSC-derived exosomes
OPCs	oligodendrocyte precursor cells
PSA	Prostate-Specific Antigen
CSCs	Cancer stem cells
CSC-exos	CSC-derived exosomes
ECSCs	esophageal carcinoma stem cell
LCSCs	lung cancer stem cells
T1DM	type 1 diabetes mellitus
Tregs	regulatory T cells
IR	insulin resistance
PBMCs	peripheral blood mononuclear cells
ET-1	endothelin 1
DR	diabetic retinopathy
PCOS	polycystic ovarian syndrome
IBD	inflammatory bowel disease
Lgr5	leucine-rich repeat-containing G protein-coupled receptor 5
ERK	extracellular-signal-regulated kinase
Mecp2	methyl CpG binding protein-2
PDGF	platelet-derived growth factor
EPCs	endothelial progenitor cells
SIRT1	sirtuin-1
CXCL12	CXC chemokine-12
Nrf2	nuclear factor E2 related factor 2
HCC	hepatocellular carcinoma
TRAIL	tumor necrosis factor-related apoptosis-inducing ligand
TRIM59	tripartite motif 5
RHPN2	rhophilin Rho GTPase binding protein 2
SEM	scanning electron microscopy
DLS	dynamic light scattering
LC-MS-MS	liquid chromatography with tandem mass spectroscopy
2DGE	two-dimensional gel electrophoresis
RT-qPCR	reverse transcriptase quantitative polymerase chain reaction
FM	fluorescent microscopy
CKD	chronic kidney disease
AIS	acute ischemic stroke
AF4	asymmetric flow field flow fractionation
